# Antiplasmodial potential of compounds isolated from *Ziziphus mucronata* and their binding to *Plasmodium falciparum* HGXPRT using biophysical and molecular docking studies

**DOI:** 10.1007/s00210-024-03611-9

**Published:** 2024-11-19

**Authors:** Kgaugelo J. Masia, Ndumiso N. Mhlongo, Ofentse J. Pooe, Mohammed A. Ibrahim, Abidemi P. Kappo, Mthokozisi B. C. Simelane

**Affiliations:** 1 Department of Biochemistry, Faculty of Science, University of Johannesburg, Auckland Park Kingsway Campus, Johannesburg, 2006 South Africa; 2 Discipline of Medical Biochemistry, School of Laboratory Medicine and Medical Science, University of KwaZulu-Natal, Durban, 4000 South Africa; 3 School of Life Sciences, University of KwaZulu-Natal, Durban, 4000 Westville South Africa; 4 Department of Biochemistry, Ahmadu Bello University, Zaria, 810107 Nigeria

**Keywords:** Betulinic acid, In silico studies, Lupeol, Methyl betulinate, *Pf*HGXPRT

## Abstract

The increasing resistance of *Plasmodium* parasites to currently available antiplasmodial therapies poses a significant challenge in treating malaria. Since ancient times, plants have served as a primary source of novel pharmacologically active compounds for drug development. Therefore, this study aimed to explore the antiplasmodial properties of pentacyclic triterpenes isolated from *Ziziphus mucronata* bark, with an emphasis on their mechanism of action. Dichloromethane and ethyl acetate extracts of the stem bark were subjected to silica gel column chromatography, which led to the isolation of three known triterpenoids: betulinic acid, methyl betulinate, and lupeol. The compounds were then evaluated for antiplasmodial activity against *Plasmodium falciparum* NF54 strains using the *Plasmodium* lactate dehydrogenase (pLDH) assay. In silico evaluation of the isolated compounds was conducted through molecular docking and further validated with in vitro experiments against a purified protein target, *Plasmodium falciparum* hypoxanthine–guanine–xanthine phosphoribosyltransferase (*Pf*HGXPRT). Betulinic acid, methyl betulinate, and lupeol exhibited potent antiplasmodial activities with IC_50_ values of 20, 10.11, and 7.56 µg/mL, respectively. Lupeol exhibited the highest binding energy of − 7.6 kcal/mol. Differential scanning fluorimetry revealed that lupeol decreases the *T*_m_ of *Pf*HGXPRT, thus decreasing the protein’s thermal stability. At high concentrations, lupeol also increased protein absorbance, indicating the detection of hydrophobic amino acids and protein unfolding. This study proves that *Z. mucronata* could serve as a reservoir of effective agents for treating malaria, while also scientifically validating its use in traditional medicine. However, further experimental studies are required to substantiate its relevant therapeutic effects.

## Introduction

Despite significant progress in malaria elimination efforts, malaria remains a major global health burden, killing hundreds of thousands annually, especially in sub-Saharan Africa (World Health Organization (WHO) World malaria report 2022). According to the World Health Organization, there were 249 million malaria cases in 85 malaria-endemic countries and 608,000 deaths attributed to malaria in the year 2022. This shows an increase relative to 2021, which recorded 244 million cases and 610,000 deaths. A large amount of the burden is borne in Africa, where the region accounts for 95% of all malaria cases and 96% of all deaths, with children and pregnant women being the most affected (World Health Organization (WHO) World malaria report 2022; Tajbakhsh et al. 2021). Therapeutic agents used to treat this disease often have numerous side effects and have been compromised by mutations in the parasite genome, leading to resistance (Sinha et al. 2014). Many of the most burdened countries, particularly those in Africa, lack access to effective malaria treatment due to several factors such as the affordability of drugs. Therefore, cost-effective antiplasmodial approaches must be employed. One promising method is to reinvestigating traditional medicines, especially plant-based remedies, to develop novel antiplasmodial drugs (Chuma et al. 2010; Nigussie and Wale 2022).

Purines are essential for cellular function, playing an important role in many metabolic and signaling processes (Campagnaro and Koning 2020). While most organisms can synthesize their purines and pyrimidines, this ability is absent in protozoans that adapted to parasitism, including *Plasmodium falciparum* (Campagnaro and Koning 2020; Chaudhary and Roos 2005). The purines of this parasite are indispensable for growth and multiplication (Quashie et al. 2010). Due to their lack of de novo purine synthesis, these parasites rely on the purine salvage pathway as an alternative. This pathway enables *P. falciparum* to acquire purine bases from the host erythrocytes, facilitating the synthesis of purine nucleotides and nucleic acids. As a result, enzymes involved in this pathway represent attractive targets for the development of novel therapeutics (Minnow et al. 2022; Soni and Pratap 2022).

In this study, the *Plasmodium falciparum* purine salvage enzyme, hypoxanthine–guanine–xanthine phosphoribosyl transferase (P*f*HGXPRT), catalyzes the reversible transfer of a phosphoribosyl group from PRPP to hypoxanthine (Hx), xanthine (Xn), and guanine (Gua), producing inosine monophosphate (IMP), xanthosine monophosphate (XMP), and/or guanosine-monophosphate (GMP), along with inorganic pyrophosphate, in a Mg^2+^-dependent reaction (Karnawat et al. 2015). The phylogenetic differences between the parasite and host enzymes provide sufficient distinctions that can be exploited for drug design (Opoku et al. 2019). Consequently, inhibiting this enzyme’s activity disrupts its ability to produce essential building blocks for DNA and RNA synthesis, ultimately leading to parasitic death, making it a promising target for the development of novel chemotherapies against malaria (Karnawat et al. 2015; Glockzin et al. 2023).

Medicinal plants play a vital role in malaria research, as several widely identified antiplasmodial agents have herbal origin. For example, quinine, isolated from the bark of the *Cinchona* tree in 1820, was the first chemically purified and effective treatment for malaria (Woodland and Chibale 2022). *Artemisia annua*, a Chinese herb first noted for its fever-reducing properties in traditional Chinese medicine, is the source of the widely used antiplasmodial agent, Artemisinin (Tse et al. 2019; Ma et al. 2020). *Ziziphus mucronata*, a member of the Rhamnaceae family, is commonly known in Zulu as “Umlahlankosi” (Mazibuko 2019). This drought-resistant species thrives in thorny areas across both tropical and temperate climates. Traditionally, root infusions and decoctions of this plant have been used to treat gonorrhea, dysentery, diarrhea, rheumatism, and snake bites (Olajuyigbe and Afolayan 2011). The dichloromethane (DCM) and ethyl acetate (EtOAc) stem bark extracts of *Z. mucronata* showed significant antiplasmodial properties, with IC_50_ values of 5.49 and 7.22 µg/mL, respectively. Therefore, bioactive compounds were isolated from these extracts and further investigated for antiplasmodial activity. Moreover, their activity against P*f*HGXPRT was explored using differential scanning fluorimetry, molecular docking studies, and ultraviolet–visible (UV–vis) spectroscopy.

## Materials and methods

### Plant collection, preparation, and extraction

The stem bark of *Z. mucronata* was collected from Rhebokfontein (27°22′44.3″S 31°28′07.3″E) within the KwaZulu-Natal Province of South Africa between February and March 2022. The plant’s stem bark was air-dried and crushed into a fine powder in preparation for sequential extraction. A 150-g sample of dried *Z. mucronata* was macerated successively with four solvents of increasing polarity. The plant was initially extracted with hexane for a week at room temperature. The solvent extraction was filtered through Whatman No. 1 filter paper and concentrated using a rotary evaporator to yield crude extracts. The crude extracts were then further macerated with DCM, EtOAC, and methanol (MeOH), thus yielding viscous crude extracts.

A bioassay-guided isolation of antiplasmodial active compounds was performed. DCM and EtOAC extracts, which exhibited high activities (IC_50_ = 5.49 and 7.22 µg/mL, respectively), were selected for further isolation of active compounds using chromatographic techniques. The crude extracts were separated by column chromatography (CC) over silica gel and eluted with a stepwise gradient. The solvent system initially contained 10% ethyl acetate in hexane, gradually increasing to 50% ethyl acetate, while collecting approximately 10–15 mL fractions. Progress in the separation of compounds was monitored by conducting thin-layer chromatography (TLC) of each fraction using hexane–ethyl acetate (7:3) solvent system and sulfuric acid in methanol for detection. Fractions with similar patterns were combined and collected as pooled fractions. The pooled fractions were then concentrated under reduced pressure to yield yellow needle-shaped powder, spindle-shaped white solid residues, and white powdery compounds, labeled as KZD60 (135 mg), KZ589 (8 mg), and KZD70 (7.56 mg), respectively. 1D ^1^H, ^13^C, and 2D distortionless enhancement by polarization transfer (DEPT) NMR studies were used to determine the chemical structures of the isolated compounds.

### Parasite cultivation and in vitro antiplasmodial activity of the compounds

The antiplasmodial activity of extracts and compounds was screened against the wild-type drug-sensitive *P. falciparum* (NF54) strain of the human malaria parasite *Plasmodium falciparum* at the H3D testing Center at the University of Cape Town, South Africa (H3D Testing Centre 2023). Continuous cultures of asexual erythrocyte stages of *P. falciparum* were maintained using the method described by Trager and Jensen (Trager and Jensen 1976), with minor modifications. The parasites were cultured in human red blood cells (O^+^, suspended at 3% hematocrit) using RPMI 1640 medium supplemented with 0.5% Albumax II, 200 M hypoxanthine, 24 µg/mL gentamicin, 25 mM HEPES, and buffered with 0.2% sodium bicarbonate (NaHCO_3_).

The in vitro antiplasmodial activity of crude extracts and isolated compounds was assessed on cultured *P. falciparum* using the *Plasmodium* lactate dehydrogenase (pLDH) assay, adapted from Makler and co-workers (Makler et al. 1993). pLDH is an enzyme that converts lactate to pyruvate, a crucial step in the glycolytic pathway of *Plasmodium* parasites. Unlike human LDH, pLDH utilizes the co-enzyme 3-acetylpyridine adenine dinucleotide (APAD) instead of NAD^+^. In the presence of phenazine ethosulfate (PES), pLDH catalyzes lactate oxidation to pyruvate while reducing APAD^+^ to APADH. This, in turn, reduces the yellow tetrazolium dye, nitroblue tetrazolium (NBT), to a dark blue-purple formazan compound. The formation of formazan is proportional to pLDH activity and can be quantified spectrophotometrically at a specific wavelength (Markwalter et al. 2016).

*Z. mucronata* crude extracts and compounds were dissolved in dimethyl sulfoxide (DMSO) to produce stock solutions of 20 mg/mL, which were then diluted with complete growth media to generate the tested concentration range. Artesunate (ARTS) and chloroquine (CQ) were used as positive controls, starting from a 1-µg/mL concentration. The stock solutions were subsequently diluted in complete media to achieve the various test concentrations. Twofold serial dilutions were made in 96-well microtiter plates in duplicate, with 40 µL of each concentration transferred to another 96-well microtiter plate. The final volume of 160 µL of parasitized red blood cells (2% parasitemia) was added to each well. The plates were incubated at 37 °C for 72 h in a sealed gas chamber with 3% O_2_ and 4% CO_2_, and the balance N_2_.

After a 72-h incubation, the wells were gently resuspended. A 15-µL sample from each well was transferred to a duplicate plate containing 100 uL Malstat reagent and 25 µL of nitroblue tetrazolium solution per well, then incubated in the dark for 20 min. The NBT solution comprised 1 and 0.05 mg/mL of NBT and phenazine ethosulfate (PES) in water. APAD^+^ (0.664 mM) was prepared by adding it to 40 mL of MilliQ water and adjusting the pH to 9 with 1 M NaOH. To the APAD solution, sodium l-lactate (0.71 M), Tris base (0.22 M), and Triton X-100 (0.2%) were added, and the solution was then diluted to 50 mL with water. The optical density was measured using a microplate reader at a wavelength of 620 nm. The concentration required for 50% inhibition of parasite growth (IC_50_) was calculated using a nonlinear regression curve in the Dotmatics software Platform.

### In vitro assessment of cytotoxicity of compounds on Chinese hamster ovary cells

A 3-(4,5-dimethylthiazol-2-yl)-2,5-diphenyl tetrazolium bromide (MTT) assay was used to assess the toxicity of the extracts and isolated compounds. Chinese hamster ovary (CHO) cells, a normal mammalian cell line, were seeded into 96-well plates (10^5^ cells/well) and incubated for 24 h at 37 °C with 5% CO_2_. The cells were then treated with various concentrations of the extracts and test compounds, ranging from 50 µM to 16 nM, and incubated for 48 h at 37 °C. Subsequently, MTT solution was added to each well after 44 h, and the plate was incubated for an additional 4 h at 37 °C. Thereafter, the medium was removed and replaced with DMSO to solubilize the MTT formazan product, and the solutions were shaken for 20 min. The optical density was measured using a spectrophotometer at a wavelength of 540 nm. The 50% cytotoxicity concentration (CC_50_) was calculated using the Dotmatics software Platform.

### Recombinant expression and purification of P*f*HGXPRT

The gene construct, pET28-a/P*f*HGXPRT, was synthesized by Genscript Corporation (Nanjing, China). As described by Opoku and colleagues (Opoku et al. 2019), chemically competent *Escherichia coli* BL21 (DE3) cells were transformed with pET28-a/P*f*HGXPRT. The positively transformed cells harboring the P*f*HGXPRT gene were inoculated in 250 mL of Luria–Bertani (LB) broth enriched with 100 µg/mL kanamycin and incubated overnight at 37 °C. The overnight inoculum was sub-cultured in 1000 mL of supplemented LB broth and incubated at 37 °C with continuous shaking until the desired optical density (OD_600_ = 0.4–0.6) was reached. P*f*HGXPRT expression was induced by the addition of 1 mM isopropyl-β-d-1-thiogalactopyranoside (IPTG). After overnight incubation, the cells were harvested by centrifugation, and the pellet fraction was suspended in lysis buffer (20 mM Tris–HCl, 500 mM NaCl, 25 mM imidazole, and 1 mM DTT at pH 7.4) on ice, followed by mild sonication. The resulting cell lysate was centrifuged at 4700 × *g* for 60 min. The P*f*HGXPRT supernatant was then collected and subjected to affinity purification.

The (His)_6_-P*f*HGXPRT protein was purified using a HisPur nickel-charged nitriloacetic acid (Ni–NTA)-immobilized metal affinity chromatography (IMAC) column, following the manufacturer’s instructions. The bound protein was eluted with phosphate buffer containing 350 mM imidazole. The pure protein was then extensively dialyzed overnight at 4 °C against phosphate buffer without imidazole. The protein fractions were analyzed using 15% sodium dodecyl sulfate–polyacrylamide gel electrophoresis (SDS-PAGE). The Bradford assay was used to estimate the protein yield, with bovine serum albumin (Sigma-Aldrich, SA) serving as the standard. Results from this section are presented in the Supplementary data (Fig. S1).

### Protein thermal shift assay using differential scanning fluorimetry

Protein thermal shift assay was conducted using the Protein Thermal Shift™ Kit (ThermoFisher Scientific). The assay, as described by Niesen et al. (Niesen et al. 2007) with slight modifications, was followed. Briefly, P*f*HGXPRT was diluted to a final concentration of 100 µg/mL (3.57 µM) in 5 µL Protein Thermal Shift™ buffer and 2.5 µL 8 × Diluted Protein Thermal Shift ™ Dye. The melting temperature (*T*_m_) was measured using a Rotor-Gene 6000 RT-PCR machine (Corbett Life Science). A temperature range of 25 to 95 °C was applied, with a ramp rate of 1 °C/min. The condition parameters were set for high-resolution melt (HRM) with excitation and emission wavelengths of 460 and 510 nm, respectively. All assays were performed both in the absence and presence of the isolated compounds at two different final concentrations: 0.5 and 1 mM for betulinic acid, methyl betulinate, and lupeol.

### Ultraviolet–visible absorption spectroscopy

UV–vis absorption spectroscopy is widely used to study proteins and their interactions with different molecules. UV–vis spectroscopy was performed using previously reported methods (Opoku et al. 2019; Salomane 2023) with slight modifications. A 35-mM solution of P*f*HGXPRT was exposed to 0.5- and 0.1-mM concentrations of betulinic acid, methyl betulinate, and lupeol for 30 min at room temperature. The spectrum readings were obtained using a UV-1800 spectrophotometer (Shimadzu, Japan) after base corrections, within the wavelength range of 200 to 600 nm. The absorption peak analysis was performed using UV Probe Software, version 2.

### Molecular docking

The binding mode of compounds from Z. *mucronata* to the P*f*HGXPRT protein structure was determined using AutoDock Tools4 (Morris et al. 2009). The crystal structure of P*f*HGXPRT was retrieved from the Protein Data Bank (PDB ID: 7TUX) (Kim et al. 2022), and ligand molecules were retrieved from the PubChem database (Hanwell et al. 2012). Energy minimization of the ligands was performed using the steepest descent method and MMFF99s, as implemented in the Avogadro program (Minnow et al. 2022). The PfHGXPRT receptor was structurally prepared using UCSF Chimera (Pettersen et al. 2004) software. AutoDock Tools were then used to determine the size of the grid box, which encapsulated the entire protein to facilitate efficient binding site identification. The grid box dimensions were set as *X* = 40, *Y* = 40, *Z* = 40, with a center at *X* = − 3.16, *Y* = − 39.361, *Z* = − 26.97, a grid spacing of 0.375 Å, and an exhaustiveness value of 10. The Lamarckian genetic algorithm, implemented in AutoDock, was used to analyze the docking of compounds to the receptor. The docked ligand conformations with the highest docking scores and an RMSD value of zero were chosen to create protein–ligand complexes. Generated complexes were visually inspected using UCSF Chimera, and the ligand–receptor interactions were presented using the LigPlot^+^ (Clarkson et al. 2004) program.

## Results and discussion

**Fig. 1:**
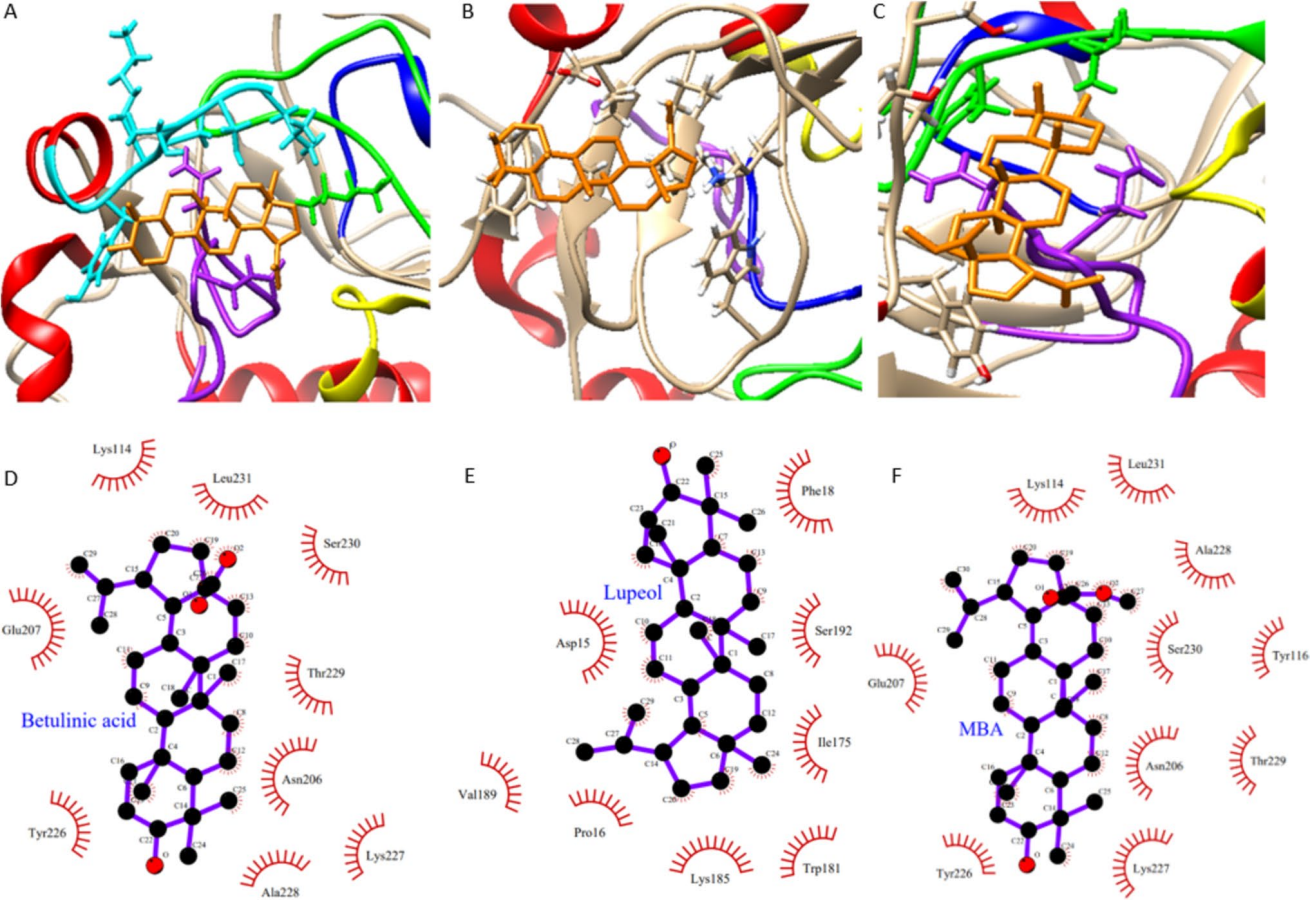
The binding modes of **a** betulinic acid, **b** lupeol, and **c** methyl betulinate ligands with P*f*HGXPRT receptor. The two-dimensional protein–ligand interaction of **d** betulinic acid, **e** lupeol, and **f** methyl betulinate with hydrophobic interactions with the sites of P*f*HGXPRT

From the bioassay-guided isolation of *Z. mucronata* bark extracts, EtAOC and DCM extracts exhibited potent antiplasmodial activity with IC_50_ values of 5.49 and 7.22 µg/mL, respectively. The extracts were fractionated and isolated by chromatographic techniques, and three known compounds were obtained (Fig. 1). One compound (K859) was isolated from the EtOAC extracts, and two compounds (KZD60 and KZD70) were isolated from the DCM extract. These were structurally elucidated to be methyl betulinate (K859), betulinic acid (KZD60), and lupeol (KZD70) by NMR spectroscopic techniques (^1^H, ^13^C, and DEPT). Table 1 summarizes the properties of the three compounds isolated from *Z. mucronata* stem bark, all of which are listed as triterpenoids.

**Table 1:**
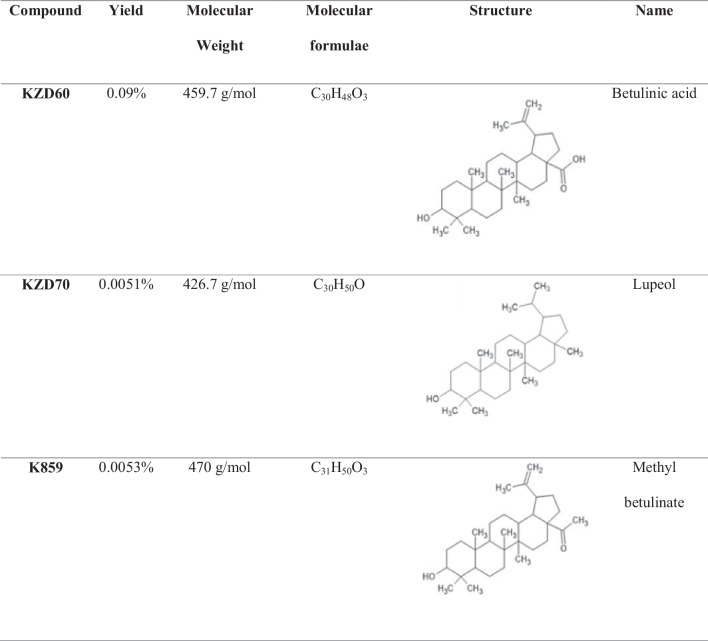
Properties of the compounds isolated from the stem bark of Z. *mucronata*

### In vitro antiplasmodial and cytotoxic activity of the isolated compounds

Four crude extracts and three major compounds were tested against the wild-type drug-sensitive *P. falciparum* strain (NF54), compared to chloroquine and artemisinin. Table 2 shows that the bark ethyl acetate and DCM extracts exhibited the highest activity and were considered potent against the *P. falciparum* strain with IC_50_ values of 5.49 and 7.22 µg/mL, respectively. Therefore, these two extracts were selected for the subsequent isolation of active compounds.

**Table 2:** In vitro antiplasmodial activity and cytotoxicity values of crude extracts and isolated compounds

Compound	Antiplasmodial activity		Cytotoxicity
	Mean (IC_50_) (µg/mL)	SEM*	IC_50_ (µg/mL)
Hexane extract	11.69	3.840	> 50
Ethyl acetate extract	7.227	1.405	10.962
DCM extract	5.494	0.034	10.018
Methanol extract	20.0	N/A	> 50
K859	10.11	1.27	> 50
KZD60	20	NA	> 50
KZD70	7.56	2.03	> 50
CQ	0.010	0.002	NA
ARTS	0.005	0.001	NA
Emetine	NA	ND	0.021

Antiplasmodial activity is defined as follows: high activity at IC_50_ $\le$ 5 µg/mL, good activity at 5–10 µg/mL, moderate activity at 15 µg/mL < IC_50_ ≤ 50 µg/mL, and inactive at 50 µg/mL. A pure compound is highly active when IC_50_ ≤ 1 µg/mL (Memvanga et al. 2015; Basco and Heseltine 2007; Alsayari and Wahab 2021). Compounds explored in this study are isolated from the stem bark of *Z. mucronata*. Traditionally, plants from this genus have played a crucial role in treating and managing various diseases, including their use as antipyretics—one of the primary symptoms associated with malaria (Singh et al. 2019). This study demonstrated that lupeol (KZD70) showed good antiplasmodial activity (IC_50_: 7.56 µg/mL), which is consistent with the findings of peer researchers (Ziegler et al. 2004), who reported that lupeol from *Ficus benjamina* also exhibited potent in vitro antiplasmodial activity. The proposed mechanism of action of lupeol is such that the compound is incorporated into the cell membrane of the host’s erythrocytes and altered, which prohibits parasite invasion as opposed to a direct toxic effect on the malaria parasite (Isah et al. 2016). Methyl betulinate (IC_50_: 10.11 µg/mL) is a naturally occurring structural analog of betulinic acid. In a study by Ziegler and colleagues (Isah et al. 2016), the antiplasmodial activity of methyl betulinate showed high activity with an IC_50_ value of 3.3 µg/mL. Other structural derivatives of betulinic acid were extensively researched (Steele et al. 1999), the majority of which possessed IC_50_ values < 10 µg/mL, further suggesting that derivatization of the compound plays a role in enhancing its antiplasmodial activity (Steele et al. 1999). Compound KZD60 (betulinic acid) showed moderate activity with an IC_50_ value of over 20 µg/mL. Similar findings were reported in a study by Kopra and co-workers (Kopra et al. 2022), where betulinic acid isolated from a Tanzanian tree, *Uapaca nitida* Mull-Arg (Euphorbiaceous), showed antiplasmodial activity translated by an IC_50_ value of 25.9 µg/mL when investigated against the K1 multidrug-resistant *P. falciparum* strain. The antiplasmodial activity of betulinic acid is similar to betulinic acid isolated from a Tanzanian tree, *Uapaca nitida* Mull-Arg (Euphorbiaceous), where the compound was evaluated against the K1 chloroquine-resistant *P. falciparum* strain, with an IC_50_ value of 25.9 µg/mL (Kopra et al. 2022). The results of the cytotoxicity study showed that the compounds were non-toxic compared to the standard cytotoxic drug, Emetine (an alkaloid), which was found to be highly cytotoxic.

### Differential scanning fluorimetry

Differential scanning fluorimetry (DSF) is a versatile technique that reports protein thermal unfolding via fluorogenic dyes (typically SYPRO Orange or ANS), often used for protein stability and protein–ligand interactions (PLI) studies (Celej et al. 2003). Ligand binding drastically affects protein stability by inducing changes in the conformation of the target protein, which, in turn, produces a given response (Acosta et al. 2020). This study explored the *Pf*HGXPRT thermal stability in the presence and absence of the study compounds at two differing concentrations (Table 3).

**Table 3:** The influence of compounds on the *T*_m_ values of *Pf*HGXPRT at varying concentrations

Compound	Concentration (mM)	*T*_m_ (°C)
P*f*HGXPRT		61.90
Betulinic acid	1	61.00
	2	61.50
Methyl betulinate	1	60.00
	2	62.20
Lupeol	1	62.20
	2	30.70

The stability of a protein is temperature-dependent due to Gibbs’ free energy during unfolding. Adding betulinic acid at both concentrations resulted in minimal changes to the observed *T*_m_ values of *Pf*HGXPRT compared to *Pf*HGXPRT alone. The presence of 0.5 mM methyl betulinate decreased *Pf*HGXPRT thermal stability by reducing the *T*_m_ by approximately 1 °C, while 1 mM increased the *T*_m_ by 2 °C, suggesting that methyl betulinate causes concentration-dependent thermal stability. *Pf*HGXPRT remained stable in the presence of methyl betulinate and betulinic acid individually. This may be due to the nature of the protein; purine phosphoribosyl transferases have oligomerization states that contribute to the protein’s overall stability (Gonçalves et al. 2022). The stability of *Pf*HGXPRT during thermal denaturation in the presence of lupeol exhibited a peculiar trend; this behavior contrasts with the typical concentration-dependent thermal stability, where higher concentrations of ligands enhance the stability of the protein. In this case, at lower concentrations of lupeol, the *T*_m_ increased slightly to 62.0 °C, while the opposite was observed at higher concentrations (1 mM), with a decrease in thermal stability resulting in a drastic change of *T*_m_ from 61.90 to 30.70 °C. This is considered a negative *T*_m_ shift, meaning lupeol caused structural changes in the protein toward a more disordered conformation (Yu et al. 2015).

### Ultraviolet–visible absorption spectroscopy

**Fig. 2:**
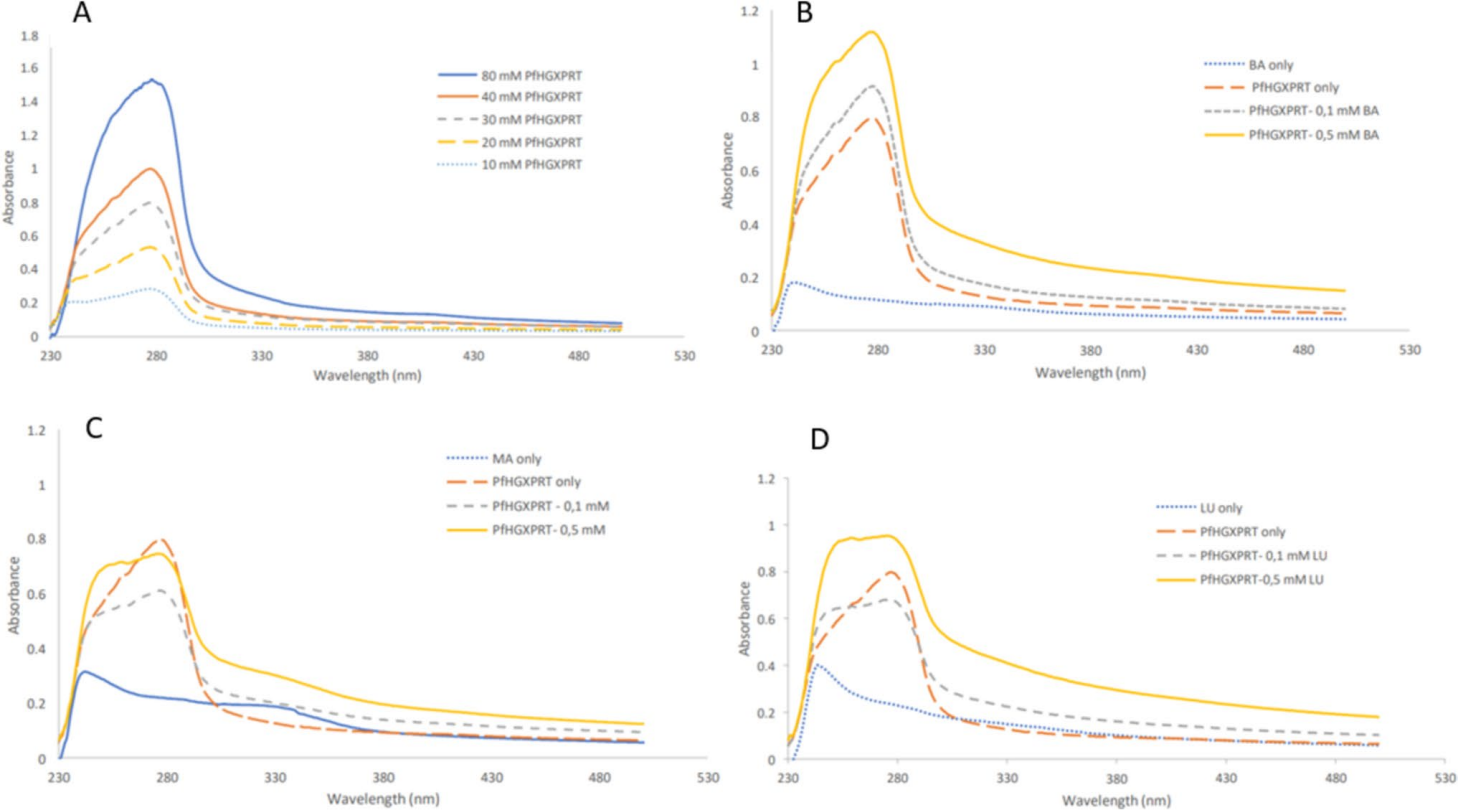
The UV–vis spectral analysis at *λ*_max_ 600 nm of P*f*HGXPRT incubated with 0.1 and 0.5 mM concentrations of **b** betulinic acid (BA), **c** methyl betulinate (MA), and **d** lupeol (LU)

The absorption spectra of P*f*HGXPRT, like many proteins, are primarily due to the aromatic amino acids. The optimum P*f*HGXPRT concentration was 30 mM (Fig. 2A). The presence of the compounds had varying influences on the absorption peak of P*f*HGXPRT at different concentrations, as shown in Fig. 2b–d. The maximum absorption peak of unbound P*f*HGXPRT was at 280 nm, mainly due to the tyrosine residues in the protein (10 tyrosine residues and 1 tryptophan residue). The phenolic hydroxyl group of tyrosine gives the residue a unique ability to interact with non-protein atoms, especially in enzymatic reactions, where it donates both an electron and a proton (Fernandes et al. 2018; Subramanyam et al. 2009). In an unbound state, tyrosine residues are unoccupied, resulting in a high absorption peak. When bioactive compounds directly interact with the protein, they influence the peak, exposing fewer or more tyrosine residues (Opoku et al. 2019). Figure 2b shows a sharp peak of P*f*HGXPRT at 280 nm, which increased in intensity as the concentration of betulinic acid increased, resulting in a hyperchromic shift. A similar effect was observed by Subramanyam and colleagues (Eftink and Pedigo 2003), where betulinic acid independently increased the intensity of the peak of human serum albumin. The absorbance of betulinic acid suggests that more tyrosine residues were exposed (Opoku et al. 2019).

Methyl betulinate (MA) had a similar effect on *Pf*HGXPRT at both concentrations (Fig. 2c). MA lowered the absorbance and shortened the wavelength, resulting in a blue shift. A study by Opoku et al. (Opoku et al. 2019) showed a similar trend, whereby the interaction of iso-mukaadial acetate and ursolic acid acetate with the same protein (*Pf*HGXPRT) decreased absorbance. The blue shift was due to the phenol rings of the tyrosine residues, which are usually buried within the hydrophobic core, being exposed during protein during unfolding (Antosiewicz and Shugar 2016). Although similar, MA had an intensified effect at low concentrations (0.1 mM). Additionally, the changes in the absorption spectra were similar to those observed in a study by Antosiewicz and Shugar (Schmid 2001), where high pH values caused protein denaturation, indicating ionization of the buried residues. This suggests that MA may cause protein denaturation in a similar manner; however, further studies must confirm this.

At both tested concentrations, lupeol caused a blue shift. However, the 0.1-mM concentration decreased the peak, while the 0.5-mM concentration had the opposite effect, increasing the intensity (Fig. 2d); these results correlate with the protein thermal shift assay. According to Eftnik and Pedigo (Antosiewicz and Shugar 2016), a blue shift indicates protein unfolding, which consequently increases the exposure of aromatic side chains to the environment. These shifts are primarily due to solvent polarity. At low concentrations, the hypochromic effect of lupeol on *Pf*HGXPRT could be due to multiple reasons, including aggregation induced by the compound, denaturation of the protein, which leads to the loss of its native structure/or changes in the local environments causing a reduction in the tyrosine absorbance at the wavelength (Cassera et al. 2011).

### Molecular docking

*Pf*HGXPRT is part of a crucial metabolic pathway in *P. falciparum* and is essential for the parasite’s survival. This enzyme displays a high affinity for all three substrates, including xanthine, which is not typically preferred by HGXPRT enzymes in most organisms. This distinction sets *Pf*HGXPRT apart from its counterpart in human red blood cells (Downie et al. 2008). Combined with its well-characterized structure and the promising results shown in previous studies (Minnow et al. 2022; Opoku et al. 2019; Roy et al. 2015; Grube et al. 2022; Karmakar et al. 2016), P*f*HGXPRT is a suitable target for this study, among other proteins. The in vitro studies using BA, LU, and MBA with *Pf*HGXPRT showed promising antiplasmodial activity. Consequently, the binding interactions of these compounds with *Pf*HGXPRT were further investigated through in silico molecular docking simulations. The binding energy and the interacting amino acid residues of *Pf*HGXPRT with each compound are shown in Table 4. The predicted binding modes of each of the compounds with *Pf*HGXPRT, along with the 2D and 3D interactions, are shown in Fig. 1. Lupeol, which exhibited the most potent antiplasmodial activity against *P. falciparum* (IC_50_ = 7.56 µg/mL), showed the best binding affinity to *Pf*HGXPRT, with a low binding energy of − 7.6 kcal/mol. This compound had hydrophobic interactions with the Phe18, Ser192, Ile175, Trp181, Lys185, Pro16, Val189, and Asp15 residues. Betulinic acid (20 µg/mL) and methyl betulinate (10.11 µg/mL) showed the same binding affinity of − 7.2 kcal/mol and, similar to lupeol, had hydrophobic interactions. Betulinic acid formed hydrophobic interactions with Lys114, Leu231, Ser230, Thr229, Asn206, Lys227, Ala228, Tyr226, and Glu207, while methyl betulinate formed interactions with residues Lys114, Leu231, Ser230, Thr229, Asn206, Lys227, Ala228, Tyr226, and Glu207.

**Table 4:** Molecular docking scores and amino acid interactions between isolated compounds and *Pf*HGXPRT

Target	Ligand	Protein–ligand interactions	
		Affinity (kcal/mol)	Interacting amino acid residues
P*f*HGXPRT	Betulinic acid	− 7.2	Lys114, Leu231, Ser230, Thr229, Asn206, Lys227, Ala228, Tyr226, Glu207
	Lupeol	− 7.6	Phe18, Ser192, Ile175, Trp181, Lys185, Pro16, Val189, Asp15
	Methyl betulinate	− 7.2	Lys114, Leu231, Ser230, Tyr116, Thr229, Asn206, Lys227, Tyr226, Glu207

Betulinic acid and methyl betulinate interacted with two amino acid residues, Glu207 and Asn206, which are part of the active loop IV of the enzyme, with the latter forming a Cα bond with Asn118, part of loop II. Loop II is involved in gatekeeping for substrate binding and product release processes (Patil et al. 2010). Methyl betulinate had an additional interaction with Tyr116, which also forms part of loop II and is predicted to interact with the ribose ring of the purine, thereby stabilizing the carbocation intermediate during enzyme catalysis (Patil et al. 2010). Lupeol docked on the protein’s outer surface, forming hydrophobic interactions with Phe18, Ser192, Ile175, Trp181, Lys185, Pro16, Val189, and Asp15 (Fig. 1). The compound primarily interacted with loop III, which contains amino acid residues 180 to 184 (Patil et al. 2010). Loop III acts as the connection between the hood and domain of the protein and is responsible for regulating catalytic events, specifically by inhibiting water molecules from entering the reaction center (Patil et al. 2010). The results suggested that the compounds could bind to the allosteric sites of the protein, as they displayed similar binding modes and shared fundamental hydrophobic interactions. The average number of hydrophobic atoms in marketed drugs is 16, highlighting the importance of hydrophobic interactions in drug design (Martínez-Peinado et al. 2021). According to Martinez-Peinado and colleagues, hydrophobic interactions occur primarily in high-efficiency ligands, making them the main driving force in drug–receptor interactions. These interactions increase binding affinity and stabilization between target–drug interfaces (Martínez-Peinado et al. 2021).

## Conclusion

The study findings showed that compounds from *Z. mucronata* elicited some activity levels against the *Plasmodium falciparum* parasite and are relatively non-toxic to normal cells. Molecular docking studies further rationalized the in vitro antiplasmodial results, examining whether the active compounds betulinic acid, methyl betulinate, and lupeol could target the P*f*HGXPRT receptor. Significant thermal shifts caused by lupeol indicate a notable impact on the overall stability of the protein. This was further corroborated by UV–vis spectral data, confirming that the compound indeed induces unfolding. Therefore, these findings demonstrate the importance of *Z. mucronata* as a valuable source in the search for bioactive compounds to treat malaria and support its use in ethnomedicinal practices. Nevertheless, further research is necessary, particularly focusing on synthesizing derivatives of the isolated compounds to enhance antiplasmodial activity and reduce cytotoxicity. Additionally, structure–activity relationship (SAR) studies will be conducted to reveal the relationship between chemical structures and their biological activity, potentially identifying key functional groups that contribute to their potency.

## Data Availability

All source data for this work (or generated in this study) are available upon reasonable request.
